# A Narrative Review of Mental Illness Stigma Reduction Interventions Among African Americans in The United States

**DOI:** 10.29245/2578-2959/2021/2.1235

**Published:** 2021-06-23

**Authors:** Kevin J. Rivera, Jenny Y. Zhang, David C. Mohr, Annie B. Wescott, Aderonke Bamgbose Pederson

**Affiliations:** 1 Department of Psychiatry, Carver College of Medicine, University of Iowa. 200 Hawkins Dr, Iowa City, Iowa, 52242, United States; 2 Department of Psychiatry and Behavioral Sciences, Feinberg School of Medicine, Northwestern University. 446 E. Ontario St, #7-200, Chicago, Illinois, 60611, United States; 3 Center for Behavioral Intervention Technologies, Department of Preventive Medicine, Feinberg School of Medicine, Northwestern University. 680 N. Lake Shore Dr, Suite 1400, Chicago, Illinois, 60611, United States; 4 Galter Health Sciences Library, Feinberg School of Medicine, Northwestern University. 320 E Superior St, Chicago, Illinois, 60611, United States

**Keywords:** African American, Black People, Intervention, Mental Health, Stigma, Stigma Reduction

## Abstract

Among African Americans, the chronicity and severity of mental illness correlates with worse health outcomes and widens health disparities. Stigma related to mental illness compounds mental health disparities by creating barriers to help-seeking behavior. We examine the current tools designed to reduce mental illness stigma and promote improved mental health outcomes among African Americans. The authors reviewed the current evidence in the literature for such stigma reduction interventions. The review team developed a focused search across four databases: PubMed, Embase, Scopus, and APA PsycINFO. Initial searches identified 120 articles, from which six studies were included as reporting on mental illness stigma reduction interventions among African Americans. We describe these four quantitative and two qualitative studies. There have been various interventions used among African Americans to reduce mental illness stigma, and the level of efficacy and effectiveness is not well studied. Our review demonstrated a need for more robust studies to yield strong evidence on effectiveness among stigma reduction interventions in this target population. The evidence does support tailoring intervention studies to this population. Effectively engaging and partnering with key stakeholders, including schools, community organizations, and faith-based institutions enhances the acceptance and delivery of stigma reduction interventions.

## Introduction

The National Institute of Mental Health reports that one in five persons in the United States experiences mental illness based on twelve-month prevalence data from 2019^1^. In the general population, mental illness remains undertreated^2-4^, and there is even lower utilization of mental health services among African Americans^5^. African Americans are less than half as likely as non-Hispanic whites to seek mental health treatment^6-8^. Despite the overall high prevalence of mental illness, stigma towards mental illness remains pervasive^9,10^. Mental illness stigma refers to negative attitudes and beliefs related to mental illness^11^. In persons with mental illness (PWMI), stigma acts as a barrier to engaging in help-seeking behavior^3,10^. African Americans are particularly vulnerable to the presence and effects of mental illness stigma^7,12-15^. Therefore, interventions that reduce stigma and increase help-seeking among African Americans with mental illness are necessary to address existing health disparities in mental health.

A well-accepted framework of mental illness stigma is founded on sociological principles, dividing stigma into the perspectives of the “stigmatizer” (individuals and society) and the stigmatized (PWMI)^10^. For society at large, stigma is enacted through stereotypes, prejudice, and discrimination that leads to a dismissal and “othering” of PWMI. For PWMI, stigma manifests through three main mechanisms: experienced, anticipated, and internalized^10^. Experienced stigma refers to the past or present encounters of PWMI with stereotyping, prejudice, and discrimination by other individuals or society^10^. Anticipated stigma occurs when PWMI expect to experience future stereotyping, prejudice, and discrimination^10^. Internalized stigma or self-stigma occurs when PWMI apply negative stereotypes and prejudice to their own self-identity^10^.

African Americans with mental illness have specific interactions with stigma which contribute to more chronic course of mental illness, greater severity at presentation and higher levels of disability^14,16,17^. Compared to non-Hispanic whites and Hispanic populations, African Americans are more likely to perceive PWMI as dangerous and to desire social distancing from PWMI^17^. When seeking mental health services, African Americans are more likely to get treatment through primary care than specialists (53% versus 32% respectively), as compared to Caucasian patients who are roughly evenly split between getting mental health care from a generalist versus a psychiatrist^7,18^. In addition, studies have also described the importance of faith-based avenues for help-seeking and community support^7,14^. Furthermore, African Americans are prone to medical mistrust of health care providers or systems, due to experienced discrimination and historical events^7^. Evidence of systemic racism exists within health care systems, including in the setting of mental health care^19,20^. Racism compounds mental illness stigma in myriad ways. African American patients are more likely to be diagnosed with schizophrenia than non-Black patients with the same cluster of symptoms^7^. Minority populations exposed to racial discrimination are more susceptible to depression, anxiety, and emotional dysregulation^16^. Mental health providers may inadvertently display racial microagressions, further driving a wedge between African Americans with mental illness and treatment engagement^7^.

Previously studied mechanisms to reduce mental illness stigma include education, social contact with PWMI including one-on-one and group contact, and social activism or “protest”^11^. While prior research has shown benefit of such stigma reduction approaches, leading experts in stigma research caution against presuming that the effectiveness of interventions as studied in one target population applies to other groups^3,21^. Given the unique context of the stigma framework among African Americans, we sought to identify and review the existing literature on stigma reduction interventions that have been examined among African Americans. This review lays a foundation upon which future interventions could be developed to be specifically effective in this population.

## Materials and Methods

### Information sources and search

The review team partnered with a research librarian to develop a focused search across four databases. The search was conducted on July 31, 2020 in the following databases: PubMed MEDLINE, Embase (Elsevier), Scopus (Elsevier), and APA PsycINFO (Ebsco). The search strategy combined database-specific controlled vocabulary and keyword terms related to mental illness stigma among African Americans and Black people^1*^. All translated searches are included in the Appendix. All databases were searched from inception to present without the use of filters or limits. All records were downloaded and underwent multi-pass deduplication in a citation management software (EndNote).

### Article selection

Titles and abstracts underwent blind screening by two independent reviewers based on inclusion and exclusion criteria as listed below. Lists of articles selected by each reviewer were then compared and reconciled based on further review of full text of each article for suitability based on inclusion and exclusion criteria. If upon further discussion, reviewers could not agree on if an article should be included in the review, a third reviewer reviewed the article and served as a tiebreaker. Selected records were then sent for data extraction.

### Eligibility criteria

**Figure 1.**
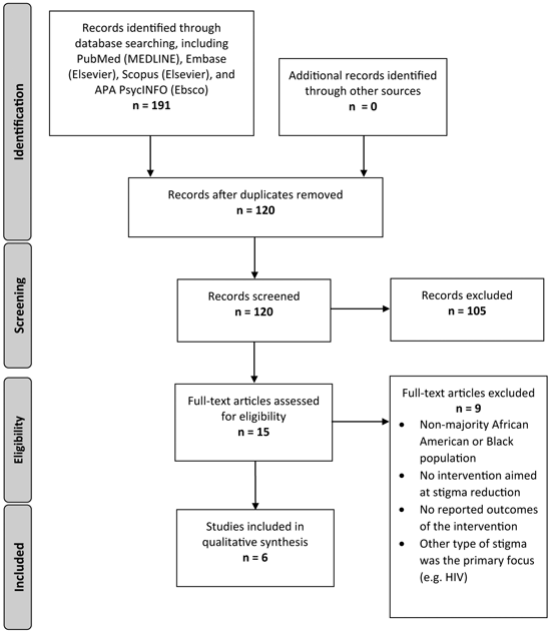
Study selection flow diagram, adapted from PRISMA^22^

Articles were included if (1) African Americans or Black persons comprised a majority (greater than 50%) or all of the study population or sample (2) the study described at least one intervention to reduce stigma among the study population, (3) the study reported on effect or outcome of the intervention. Articles were excluded if (1) the study did not address mental health, (2) the study’s primary focus was on non-Black or non-African American populations, (3) the study focused on other stigmatizing health conditions like HIV infection. See Figure 1 for a flow diagram for article selection.

### Data extraction

Based on initial review, the studies were deemed too heterogeneous to perform standardized data extraction. Thus, we present a review of each study with discussion of pertinent themes as they apply to mental illness stigma reduction in African American or Black people.

## Results

Among the 120 articles identified by initial search parameters, six articles (Table 1) met study criteria. Four articles were quantitative and two were qualitative. The six titles included in our narrative review detail distinct interventions to reduce mental illness stigma among populations that were composed of mostly or exclusively African American persons. Two church-based studies did not report the specific racial demographics of their samples, though the populations of church congregants were described as mostly African American. All study samples included adults only. All described initiatives implemented in the United States, but represented various geographic regions including the West, Midwest, Northeast, and Southeast. While the studies vary in their specific target populations, scope, types of intervention, outcome measures, analytic methods, and findings, they all lacked methodological rigor. Three studies were very small, enrolling less than 50 participants. Table 1 summarizes the study characteristics and key contributions to the current understanding of mental illness stigma reduction specifically among African American persons. Below, we review each paper in more detail.

### Distributing a psychoeducational booklet based on African American mental health consumer experiences to new mental health clients

Alvidrez et al. (2009)^23^ assesses a stigma psychoeducation intervention developed to increase mental health treatment utilization among African Americans with mental illness. This intervention was based in part on findings reported in a separate qualitative interview study (Alvidrez et al., 2008)^29^, wherein African American consumers of community mental health services in San Francisco endorsed having found ways to cope with mental illness stigma. These strategies fell broadly into one of two categories: attitudes or behaviors. Attitudes included prioritizing health above what others think, normalizing mental health problems, and feeling proud of seeking help. Behavioral strategies to reduce stigma included relying on social support; most participants relied on an established network whereas fewer reported finding a new support network. Another behavioral strategy was to control with whom they would share information about their mental health treatment as well as how much information they would share with those individuals. In Alvidrez et al. (2009)^23^, authors describe using the consumer-based stigma reduction strategies above to develop a psychoeducational booklet titled “Getting Mental Health Treatment: Advice from People Who’ve Been There”. This study enrolled a sample of 42 new African American clients who had completed intake evaluations and were offered services at a county hospital-based mental health clinic. Primary outcomes for this study were listed as perceived helpfulness of the information provided, treatment entry, treatment attendance, and perceived stigma. Upon entry into the study, participants’ baseline concerns about mental health treatment were measured using a 12-item scale developed by the study authors. Other measures included whether participants entered into individual or group therapy, how many therapy visits they attended during a 3-month follow up period, and scores on the Devaluation-Discrimination Scale^30^ which assesses beliefs about the stigmatization of mental illness; this was administered at baseline and 3 month follow up. 22 participants were randomly assigned to receive the psychoeducational booklet and 20 received two brochures with general information about county mental health services and clinic services. In both groups, clients were given the materials, which were also read aloud to them; these informational sessions lasted about 15-30 minutes.

The results of Alvidrez et al. (2009)^23^ did not show significant differences in any of the primary outcomes, including helpfulness of the information received, rates of treatment entry, or in the mean number of therapy sessions attended. The type of material received did not have a statistically significant impact overall on the beliefs about stigmatization at 3 months. The authors did discuss additional exploratory outcomes, finding that clients’ treatment concerns at baseline mediated significant effects on stigma. For individuals with higher perceived need for treatment and for those who reported feeling more uncertain about the process and benefits of treatment at baseline, participants who received the psychoeducation booklet had lower stigma at 3 months than those who received general information brochures. The converse was true: among those with lower perceived need for treatment and lower uncertainty at baseline, receiving the general information brochures was associated with decreased stigma. Thus, a psychoeducational booklet developed specifically for African Americans did not impact treatment entry, but it may have been more effective at reducing stigma among those who felt stronger about their need for treatment and those who felt less familiar with what treatment entailed. However, like several of the studies that will follow in this review, this study had significant limitations due to small sample size, and it may not have been adequately powered for its analyses. The possibility of type I error for any significant differences should be considered, and it is important to highlight that the intervention was not superior to control for any of the primary outcomes.

### Comparing psychoeducational programs based on contextual versus biomedical models of depression for majority African American community members

Rusch et al. (2010)^24^ proposed that a stigma reduction intervention based on a contextual model of depression, which emphasizes the role of environmental factors, would be more effective at decreasing stigma than an intervention based on a biomedical model, which presents depression as a medical disease. 115 participants with self-reported depressive symptoms were recruited from three community organizations which provide services to low-income community members. 80.9% of study participants were African American. Participants were non-randomly assigned to either a contextual anti-stigma program or a biomedical anti-stigma program, both about 25 minutes in length, and were administered in smaller groups. They were adapted from previously-developed programs and involved a common initial discussion of depression using digital presentation slides with voiceover narration, brief interview with a depression expert, and sixteen video clips of individuals sharing their experiences with depression. The programs then varied in how they presented information on the causes and treatments of depression; the contextual program highlighted how difficult events and stressors can contribute to depression and discussed psychotherapeutic treatment approaches, whereas the biomedical program described depression as a result of neurotransmitter imbalances with biologic and genetic influences and focused on pharmacologic treatment approaches. Neither program was designed for cultural specificity. There was no control group in this study. The main hypothesis tested in this study was that the contextual program would decrease stigma more than the biomedical program. Before each program, participants completed the Center for Epidemiologic Studies Depression Scale (CES-D)^31^ which measures depressive symptoms, as well as the Depression Self-Stigma Scale (DSSS)^32^, which includes subscales measuring general self-stigma and treatment-seeking stigma. Higher scores on both scales indicate higher severity. Immediately following the program, participants repeated the DSSS and completed a 4-item program believability questionnaire. At 2-month follow up, participants repeated the CES-D and DSSS.

There were no significant differences between the two groups in depressive symptoms or stigma at baseline, and there was no significant difference between the programs in believability. The groups were analyzed by both condition and time, with the DSSS general self-stigma and treatment-seeking stigma subscales analyzed separately. There were no significant differences in general self-stigma scores according to condition, time, or in the condition by time interaction. For treatment-seeking stigma, scores were unchanged for those in the contextual group. Scores for those in the biomedical group increased at first follow up and remained above baseline at 2-month follow up, but further analysis showed that the biomedical group showed higher stigma than the contextual group only at immediate follow up. Baseline depressive symptoms did not mediate any effects. In summary, in this intervention comparison study conducted among a majority African American sample, there was no evidence supporting the study’s primary hypothesis that a short intervention based on a contextual model of depression was more effective at reducing stigma than one based on a biomedical model of depression, and neither intervention was demonstrated to significantly reduce stigma over time. This study was one of the larger studies in this review, but it was limited by non-randomized sorting of participants and lack of control group.

### Implementing a comprehensive training curriculum for African American churches

The Promoting Emotional Wellness and Spirituality (PEWS) program described in Williams et al. (2014)^8^ was developed with the goals of educating clergy on the signs and symptoms of depression, reducing stigma associated with depression, and promoting treatment-seeking for depression. This program initially brought local faith leaders, mental health professionals, mental health consumers, and community members together over the course of two annual “Spirituality and Wellness” conferences. The conferences led to the development of the PEWS training curriculum, designed as a four-day, 10-hour training which sought to help churches develop a mental health ministry committee or expand their existing health ministry to include a mental health component. The curriculum includes a pretest to assess baseline knowledge, video vignettes of people who have received mental health treatment, video presentations in which pastors highlight the connection between mental illness and spirituality, psychoeducation on depression and its treatment, an introduction to effective communication techniques, crisis intervention skills training, recommendations for referring congregants to higher levels of care, and a posttest to assess retention of training material. While pretests and posttests were a part of the curriculum, these were not designed for research purposes, and the results were not reported.

The paper describes the key takeaways after implementing the PEWS training curriculum in two majority African American Baptist churches in urban cities in New Jersey: one medium-sized church (approximately 400 congregants) and one megachurch (approximately 2000 congregants). The churches are described as approximately 98% African American, though racial demographics of the sample is not reported. At the medium-sized church, 26 individuals attended the training, which was delivered over the course of two weeks. As a result of this training curriculum, church members started a Health and Wellness Ministry Committee, composed of nine members. This ministry went on to invite health professionals to speak at their annual health fair, referred congregants to local mental health providers, and started a Bereavement Support Group. At the megachurch, 70 individuals attended the training, delivered over the course of four weeks. After the training was implemented, the megachurch added a Mental Health Committee to their Nurses Auxiliary, which had previously offered programs for those with physical health problems. This committee went on to sponsor several events featuring mental health professionals dealing with depression, the relationship between physical and mental health, parenting skills, and stress management. The authors describe some key “lessons learned,” including the centrality of a community-based participatory research approach in developing the PEWS training curriculum, the importance of developing relationships with church leaders in order to generate interest in the PEWS training curriculum and ownership of the resulting initiatives, and the necessity of building some flexibility into this training curriculum to adapt to church schedules and availability. As a qualitative study, this study offers a descriptive evaluation of the outcomes of intervention but is limited in its ability to demonstrate a measured stigma reduction among its participants or the churches studied.

### Comparing video and in-person delivery of a contact intervention for African American undergraduate students

Vinson et al. (2016)^26^ compared two delivery methods for a contact stigma reduction intervention: in-person and video. Out of a total sample of 158 African American undergraduate students in the southeastern United States, 96 participants were non-randomly assigned to an in-person group in which an African American man shared about his experience with panic disorder, his decision to seek treatment, his experience in therapy, reactions of his support network, and his recovery. The presentation lasted 12 minutes and did not include a question and answer or discussion portion. This presentation was recorded, and later, the remaining 62 participants watched this recording for the video group.

Immediately before, immediately after, and 2 weeks after the interventions, participants completed questionnaires measuring stigma and attitudes toward seeking therapy. The Social Distancing Scale^33^ measures willingness to socialize with PWMI, and the Attribution Questionnaire 20^34^ measures attitudes and beliefs about individuals with mental illness. The Thoughts About Therapy Scale (TAPS)^35^ measures apprehension toward seeking therapy and experiences in therapy, and the Inventory of Attitudes Toward Seeking Mental Health Services^36^ assesses indifference to stigma, help-seeking propensity, and psychological openness. There were significant improvements on all measures for both groups overall, but there was no significant difference between in-person and video delivery. There was no group by time interaction. For the whole sample, desire for social distance and negative attributions about people with mental illness significantly decreased immediately following the interventions. This effect was sustained at 2 week follow up for social distance, but follow up scores for negative attributions were no different compared to pretest scores. TAPS scores decreased between pretest and posttest, and this effect was sustained at follow up. Favorable attitudes toward help-seeking significantly increased from pretest to posttest, but attitudes decreased at follow up to a level no different from pretest. This study suggests that, among African American college students, brief in-person and video contact interventions may reduce some aspects of stigma for a short period of time. Limitations include the lack of control group and randomization which would otherwise reduce the possibility of type I error. Additionally, with a short follow up period, the durability of the effects shown is not known.

### Providing psychoeducation through film and discussion in an African American church

Garner and Kunkel (2020)^27^ report on a quality improvement project implemented at one majority African American church in the southeastern United States which had, over the course of one year, experienced nine suicide fatalities, attempted suicides, and inpatient psychiatric hospitalizations among its congregants. Study authors devised a multifaceted intervention for ministerial staff with the primary aim of increasing education and engagement on the importance of screening, referral, and follow up of congregants with increased risk for depression and suicidality. The secondary aim was mental illness stigma reduction. The intervention included the following components: screening a one-hour documentary film “Shadow Voices: Finding Hope in Mental Illness,” training in the use of the Patient Health Questionnaire-9, and open forum for discussion and questions. Study authors also created a community resource binder which listed local behavioral health providers and agencies to aid in referrals to treatment by the pastoral staff. Response to this intervention was measured by a Mental Health Knowledge test (MHKT; not described in the study) and the Community Attitudes Toward Mental Illness Scale (CAMI)^37^, which is a 40-item scale with subscales for the following: community mental health ideology, benevolence, authoritarianism, and social restrictiveness. The CAMI was developed and validated for subscale analyses. The MHKT and CAMI were administered immediately prior to the intervention and at 90 days post-intervention.

Twenty-eight members of the faith-based institution’s ministerial team participated in the intervention; racial and ethnic background of the study participants were not reported, but the church was described as mainly African American. There was no control group in this study. Results showed a significant increase in scores on the MHKT at follow up. For the CAMI, there was no significant change in the benevolence and ideology subscales, which represent acceptance of people with mental illness, but there was a significant reduction in the authoritarianism and social restrictiveness subscales. In this pretest-posttest design study, an intervention delivered to a church’s pastoral staff was associated with increased mental health knowledge and possibly decreased stigma 3 months post-intervention. However, this study showed significant limitations in its design and analysis. With a very small sample and lack of control group, the possibility of type I error with the should be carefully considered for any of the associated differences seen on the CAMI subscales.

### Involving Black transition-aged students in a participatory action research project

A dissertation by Hargrove (2020)^28^ examined whether mental illness self-stigma could be reduced among Black transition-aged youth through engagement in a semester-long participatory action research (PAR)-informed project. PAR is a collaborative model in which formally trained researchers partner with community members who become both study participants and co-researchers. The model was developed with the purpose of generating social change through a process by which participants investigate a common shared problem and later develop an action component which has direct impact on the community of which the participant co-researchers are a part. The primary investigator recruited participant co-researchers who identified as Black (excluding biracial and multiracial persons) and had either been born in the U.S. or arrived in the U.S. before age 6; they also met inclusion criteria if they were between ages 18 to 25 and had an active recognized mental health challenge. Seven participant co-researchers, all students from the University of Massachusetts Boston, composed the study sample, and data was gathered using grounded theory qualitative interviews. The primary investigator reports gathering additional data through process notes from weekly group sessions, as well as several questionnaires measuring internalized racism, self-stigma, and self-esteem as it relates to racial and ethnic identity, though the results of these questionnaires were not reported in the dissertation. Participant co-researchers started the project with a two-day orientation, followed by weekly 2.5 hour sessions during which the group developed research questions and then designed a study which they would implement The PAR project lasted one semester.

The result of the primary researcher’s study was a theoretical model called the PAR Process of Change, which conceptualized the process by which participant co-researchers experienced a reduction in mental illness stigma. The PAR Process of Change included five stages: Joining, Establishing Structure and Safety, Opening Up and Receiving Support, Discovering and Creating Community, and Facilitating Change. The dissertation includes several excerpts from the qualitative interviews which support this model and display the participant co-researchers’ reported changes in attitude and reflections on their experienced stigma reduction. This qualitative study highlights an interesting, intensive, and comparable longer-term intervention designed to both reduce mental illness stigma and generate social change. However, with its small a very specific sample, lack of quantitative results, and theoretical outcome, the study’s conclusions are limited in terms of replicability and generalizability.

## Discussion

Given the well-established inequities African Americans face in terms of mental illness, treatment, and the stigma associated with these, the collection of evidence included in this review is important to highlight. The common theme among the included studies is the shared sense that stigma reduction efforts need to be culturally informed and tailored to apply to African Americans. Indeed, the sentiment that effectiveness of interventions in one group may not be generalizable to other groups has been advocated for by experts in the field^3,21^, indicating the importance that stigma reduction measures studied in other populations be examined for efficacy in African American populations. Existing stigma reduction interventions studied in other populations can broadly be categorized as psychoeducation-, contact-, or protest-based^11^. While some of the studies included in this review describe developing novel interventions altogether, some studies do take existing psychoeducational or contact-based materials made for a broader audience and focus on targeted delivery of these to African American communities. Most of the studies describe group-based interventions, however Alvidrez et al. (2009)^23^ describes a facilitated one-on-one intervention. Settings varied from schools to community-based organizations to churches. Lengths of interventions include a 12-minute contact intervention, a 10-hour training curriculum, and a semester-long participatory research project. With such variation in design, the collection of these studies demonstrates that there is no current consensus on a single most effective intervention to reduce stigma in this population, which invites innovation in design among practitioners and offers areas of future study, such as length of intervention.

While previous reviews of stigma reduction literature have focused on types of interventions, other strategies have been to examine interventions targeting specific forms of stigma, such as internalized stigma, experienced stigma, or anticipated stigma^10^. Though some studies in this review use these terms for describing their interventions, it is not a universal feature. As more research is done in this population in the future, applying these frameworks may enhance the ability to compare and aggregate data. Future researchers may also look to some of the validated stigma-measuring scales used in the papers here (Alvidrez et al., 2009; Rusch et al., 2010; Vinson et al., 2016) ^23,24,26^ to consider whether there can be greater concordance in future measures.

Furthermore, African Americans should not be viewed as a monolithic group. Each of the particular findings in any of the studies included in this review may still have limited generalizability, given that each study’s population varied demographically from others in terms of age, socioeconomic status, region, education, and religious context. Even these demographic categories might overlook subtle nuances in how persons within the population respond to the content or style of a planned intervention, such as how baseline attitudes toward mental illness and treatment might impact one’s receptiveness to a tailored intervention^23^.

As progress is made toward developing effective mental illness stigma reduction interventions among African Americans, more research can be done on effective delivery methods and settings. For example, among African American college students, a video-based contact intervention was found to be equally effective compared to an in-person intervention with the same content, raising the possibility that video may help future efforts to scale the delivery of certain stigma reduction interventions. Delivery setting is also important. As evident in two of the papers^26,27^, the tendency to seek help for mental health problems through faith-based mechanisms is important to many African Americans with mental illness. The active role of collaboration between mental health providers and faith-based institutions is a key area of study with the potential for promising results especially given the impact of medical mistrust on help-seeking behavior in traditional health settings^19,20,38^. In such collaborations, psychoeducation and concurrent engagement of faith-based collaborators is crucial. Other studies included in this review delivered interventions in university and community organization sites, with only one^23^, choosing a clinical setting for the intervention. This highlights the importance of researchers partnering with key community stakeholders in designing and testing their interventions. Savage et al. (2013)^39^ provides an example of a large-scale project involving the partnership of non-profit organizations, churches, community mental health centers, a state department of mental health, and a university in order to increase mental health service utilization and reduce mental illness stigma in a rural, predominantly African American region of Alabama. While resource-intensive, such partnerships may influence the design of stigma reduction interventions such that they may be more feasibly implemented in a variety of community settings.

There are several limitations of our narrative review, which comprises descriptions of a small number of studies which, due to their heterogeneity, cannot be subjected to in-depth comparative analysis. The quality of the evidence in the included studies is generally limited. Among the four quantitative studies, samples were small and generally lacked methodological rigor, with only one using a randomized control design^23^. While Vinson et al. (2016)^26^ found that the video contact intervention had comparable effect to an in-person intervention on stigma measures for African American undergraduates, this was a non-randomized non-controlled study, so the overall effectiveness of these as stigma reduction interventions is unclear. The importance of the need for rigorous evaluation and analysis of stigma reduction interventions is highlighted in Rusch et al. (2010)^24^, which provides at least one example of a type of intervention which may actually increase stigma for a short period. In this study, framing depression as an illness due to genetic and biochemical dysregulation had an immediate negative effect, though this approach showed no long-term difference on stigma when compared to framing depression as an illness dependent on environmental factors^24^. While usually well-intentioned, interventions to reduce stigma in this population must be carefully evaluated before they can be widely recommended or implemented. Other limitations include the lack of available literature reporting on stigma reduction efforts among African American minors or among non-English speaking Black people. Additionally, all studies included in this review were based in the United States, so generalizability of our findings may not extend to other country settings.

Future studies among African Americans and other Black persons in the United States should ideally employ control groups and validated clinical scales for quantifying or qualifying stigma. In addition, developers of future mental illness stigma reduction interventions should consider the cultural, social, and political context specific to African Americans, including factors of medical mistrust of the health care system, the impacts of interpersonal and structural racism on mental health, and the ways in which racial prejudice and discrimination interact with mental illness stigma.

## Conclusions

This narrative review reports on strategies to reduce stigma among African Americans based on the current literature. There are a limited number of studies describing interventions for mental illness stigma reduction among African Americans. Literature search by our parameters yielded six articles meeting study criteria. Out of the six studies included in this review, four are quantitative and two are qualitative. Among these studies, implementing a variety of interventions, such as psychoeducation through tailored booklets, group teaching sessions, video and in person contact interventions, and involvement in longer-term participatory research were feasible and acceptable to study participants, but results were generally short of meeting broad goals of significant stigma reduction. While there were demographic differences among the populations studied, there were some common elements between studies; for example, five of the studies described interventions delivered in non-clinical community-based sites, such as faith-based institutions and university settings. There were several limitations including a small number of studies, heterogeneity among the studies, and poor methodological quality. However, this review demonstrates examples of successful implementation, suggests possible areas of future inquiry, and underscores the need for high quality study design. While the body of evidence is still in its early stages, the examples provided in this review may offer helpful direction for future development. As the attention toward racial inequities in mental health increases, addressing mental illness stigma among African Americans must remain a priority.

## Figures and Tables

**Table 1.** Characteristics of stigma reduction intervention papers

Reference	Population and location	Sample size	Sample or population demographics	Study type	Intervention description	Comparison	Variables	Pertinent findings on stigma reduction
Alvidrez et al. (2009)^23^	Clients of one county hospital-based mental health clinic, San Francisco	42	100% African American sample	Quantitative - randomized control	Psychoeducational booklet based on African American mental health consumers’ experiences	General information brochures describing services	Treatment entry, therapy attendance, perceived stigma	There was no overall difference in treatment entry, treatment adherence, or perceived stigma. For those with greater perceived need for treatment or greater uncertainty about treatment, the psychoeducation booklet reduced stigma. General information brochures reduced stigma for those with less interest in or less concern about treatment.
Rusch et al. (2010)^24^	Individuals with depressive symptoms receiving services through community organizations, Wisconsin	115	81% African American sample	Quantitative - nonrandomized comparison	Anti-stigma program which presents a contextual model of depression	Anti-stigma program which presents a biomedical model of depression	General self-stigma, treatment-seeking stigma	Groups did not differ for general self-stigma. For treatment-seeking stigma, there was an increase in stigma with the biomedical program directly following the intervention, but there was no difference between the groups at two months.
Williams et al. (2014)^25^	Congregants of African American Baptist churches, New Jersey	96	98% African American church population	Qualitative - case study	Training curriculum with video vignettes, psychoeducation, and training in communication and crisis intervention	None	Stigma reduction through the development of church-based mental health ministry committees	The PEWS training curriculum engaged congregants at one medium-sized church and one megachurch, which led to the development of mental health committees, both of which invited mental health professionals to interface with congregants at health promotion events.
Vinson et al. (2016)^26^	Undergraduate students, southeastern United States	158	100% African American sample	Quantitative - nonrandomized comparison	Video contact intervention	In-person contact intervention	Desire for social distance from people with mental illness, negative attributions about people with mental illness, apprehension toward therapy, attitudes toward help-seeking	There were no significant differences between video and in-person contact interventions. For both interventions, all measures improved immediately following the interventions, but the durability of these effects were mixed two weeks later.
Garner and Kunkel (2020)^27^	Ministerial staff of one non-denominational church, southeastern United States	28	Majority African American church population	Quantitative - pretest/posttest	Educational session including watching a documentary film, an introduction to PHQ-9, open discussion forum	none	General mental health knowledge and negative attitudes toward individuals with mental illness	General mental health knowledge increased and was retained. Positive attitudes toward people with mental illness did not change, but negative attitudes decreased.
Hargrove (2020)^28^	Transition-aged youth enrolled as undergraduate students, Boston	7	100% Black sample	Qualitative - grounded theory	Semester-long participatory action research-informed project	None	Mental illness stigma and the process for change in stigma	Study participants self-reported reductions in mental illness stigma. Grounded theory interviews informed a theoretical explanatory model for how a PAR-informed project changed participants' stigmatizing beliefs.

## Footnotes

1* While the total population of Black persons in the United States include some groups that do not identify as African American, we use the term African American throughout most of this review to be consistent with the terminology found in most of the papers included for review as well as previous studies which explore stigma in African Americans. Exceptions are made in discussing papers which specifically provide rationale for their use of Black persons in defining their target population.

## Appendix: Translated searches for each database

### Ovid MEDLINE

("African Continental Ancestry Group"[Mesh] OR African American*[tiab] OR African immigrant*[tiab] OR Black[tiab] OR BIPOC[tiab] OR latino*[tiab] OR Latina*[tiab] OR latinx[tiab]) AND ("Mental Disorders"[Majr] OR "Mental Health"[Majr] OR "Mental Health Services"[Majr] OR "mental health"[ti] OR "mental hygiene"[ti] OR "mental wellbeing"[ti] OR "mental illness*"[ti] OR "mental disorder*"[ti] OR "mental disease*"[ti] OR psych*[ti] OR "behavioral therap*"[ti] OR "behavioral medicine"[ti] OR depress*[ti] OR schizophren*[ti] OR schizoaffect*[ti] OR bipolar[ti] OR anxiety[ti] OR obsessi*[ti] OR phobi*[ti] OR panic[ti] OR "personality disorder*"[ti] OR suicid*[ti]) AND ("stigma reduction"[tiab] OR "discrimination reduction"[tiab] OR "stigma intervention*"[tiab] OR "discrimination intervention*"[tiab] OR "reducing stigma*"[tiab] OR "reduce stigma*"[tiab] OR "reduces stigma*"[tiab] OR "reduced stigma*"[tiab] OR "reducing discrimin*"[tiab] OR "reduce discrimin*"[tiab] OR "reduces discrimin*"[tiab] OR "reduced discrimin*"[tiab])

### Embase

(((('black person'/exp OR 'hispanic'/exp OR african) AND american*:ti,ab OR african) AND immigrant*:ti,ab OR black:ti,ab OR bipoc:ti,ab OR latino*:ti,ab OR latina*:ti,ab OR latinx:ti,ab) AND ('mental disease'/exp OR 'mental health'/exp OR 'mental health service'/exp OR 'mental health':ti OR 'mental hygiene':ti OR 'mental wellbeing':ti OR 'mental illness*':ti OR 'mental disorder*':ti OR 'mental disease*':ti OR psych*:ti OR 'behavioral therap*':ti OR 'behavioral medicine':ti OR depress*:ti OR schizophren*:ti OR schizoaffect*:ti OR bipolar:ti OR anxiety:ti OR obsessi*:ti OR phobi*:ti OR panic:ti OR 'personality disorder*':ti OR suicid*:ti) AND ('stigma reduction':ti,ab OR 'discrimination reduction':ti,ab OR 'stigma intervention*':ti,ab OR 'discrimination intervention*':ti,ab OR 'reducing stigma*':ti,ab OR 'reduce stigma*':ti,ab OR 'reduces stigma*':ti,ab OR 'reduced stigma*':ti,ab OR 'reducing discrimin*':ti,ab OR 'reduce discrimin*':ti,ab OR 'reduces discrimin*':ti,ab OR 'reduced discrimin*':ti,ab)

### Scopus

TITLE-ABS (“African American*” OR “African immigrant*” OR Black OR BIPOC OR latino* OR Latina* OR latinx)

AND

TITLE ("mental health" OR "mental hygiene" OR "mental wellbeing" OR "mental illness*" OR "mental disorder*" OR "mental disease*" OR psych* OR "behavioral therap*" OR "behavioral medicine" OR depress* OR schizophren* OR schizoaffect* OR bipolar OR anxiety OR obsessi* OR phobi* OR panic OR "personality disorder*" OR suicid*)

AND

TITLE-ABS ("stigma reduction” OR "discrimination reduction" OR "stigma intervention*" OR "discrimination intervention*" OR "reducing stigma*" OR "reduce stigma*" OR "reduces stigma*" OR "reduced stigma*" OR "reducing discrimin*" OR "reduce discrimin*" OR "reduces discrimin*" OR "reduced discrimin*")

### PsycINFO

(DE "Blacks" OR DE "Latinos/Latinas" OR DE "Mexican Americans" OR “African American*” OR “African immigrant*” OR Black OR BIPOC OR latino* OR Latina* OR latinx)

AND

(DE "Mental Health Programs" OR DE "Mental Health Services" OR DE "Mental Disorders" OR DE "Mental Health Stigma" OR DE "Affective Disorders" OR DE "Anxiety Disorders" OR DE "Bipolar Disorder" OR DE "Psychosis" OR "mental health" OR "mental hygiene" OR "mental wellbeing" OR "mental illness*" OR "mental disorder*" OR "mental disease*" OR psych* OR "behavioral therap*" OR "behavioral medicine" OR depress* OR schizophren* OR schizoaffect* OR bipolar OR anxiety OR obsessi* OR phobi* OR panic OR "personality disorder*" OR suicid*)

AND

("stigma reduction” OR "discrimination reduction" OR "stigma intervention*" OR "discrimination intervention*" OR "reducing stigma*" OR "reduce stigma*" OR "reduces stigma*" OR "reduced stigma*" OR "reducing discrimin*" OR "reduce discrimin*" OR "reduces discrimin*" OR "reduced discrimin*")
